# Management of drug-induced liver injury in people with HIV treated for tuberculosis: 2024 update

**DOI:** 10.4102/sajhivmed.v25i1.1558

**Published:** 2024-03-30

**Authors:** Tom Boyles, Rebecca H. Berhanu, Neliswa Gogela, Hannah Gunter, Tamsin Lovelock, Ndiviwe Mphothulo, Arifa Parker, Helena Rabie, Lauren Richards, Phumla Sinxadi, Camilla Wattrus, Mahomed-Yunus Moosa

**Affiliations:** 1 Clinical HIV Research Unit, Helen Joseph Hospital, Johannesburg, South Africa; 2 Right to Care (NPC) Centurion, Johannesburg, South Africa; 3 London School of Hygiene and Tropical Medicine, London, United Kingdom; 4 Department of Medicine, Division of Infectious Diseases, Vanderbilt University Medical Centre, Nashville, Tennessee, United States of America; 5 Division of Hepatology, Department of Medicine, Faculty of Health Sciences, University of Cape Town and Groote Schuur Hospital, Cape Town, South Africa; 6 Division of Clinical Pharmacology, Department of Medicine, Faculty of Health Sciences, University of Cape Town and Groote Schuur Hospital, Cape Town, South Africa; 7 Division of Infectious Diseases, Department of Medicine, Faculty of Medicine and Health Services, Stellenbosch University and Tygerberg Hospital, Cape Town, South Africa; 8 Southern African HIV Clinicians Society, Johannesburg, South Africa; 9 Unit for Infection Prevention and Control, Department of Medicine, Stellenbosch University and Tygerberg Hospital, Cape Town, South Africa, South Africa; 10 Division of Infectious Diseases, Department of Medicine, Stellenbosch University and Tygerberg Hospital, Cape Town, South Africa; 11 Department of Paediatrics and Child Health, Stellenbosch University and Tygerberg Hospital, Cape Town, South Africa; 12 Division of Infectious Diseases, Department of Internal Medicine, Faculty of Health Sciences, University of the Witwatersrand, Johannesburg, South Africa; 13 SAMRC/UCT Platform for Pharmacogenomics Research and Translation, South African Medical Research Council, Cape Town, South Africa; 14 Department of Infectious Diseases, Division of Internal Medicine, Nelson R Mandela School of Medicine, University of KwaZulu-Natal, Durban, South Africa

## Aim and scope

In 2013, the Southern Africa HIV Clinicians Society (SAHCS) published a consensus statement on the management of drug-induced liver injury (DILI) in people with HIV treated for drug-sensitive tuberculosis (TB).^1^ This publication represents the first update to that statement. A group of experienced clinicians from the fields of infectious diseases and HIV medicine, clinical pharmacology, and hepatology was convened. Online meetings and email communication were used to discuss and finalise the content of the guideline. The evidence base for providing guidance remains limited, so some recommendations are based on expert opinion. Our primary aim is to provide practical guidance to healthcare workers on the diagnosis and management of adults and children with abnormal liver function tests (LFTs) while taking drug-sensitive anti-tuberculous therapy. For patients on rifampicin (RIF)-resistant anti-tuberculous therapy, specialist guidance should be sought.

## Background

In 2022, South Africa (SA) had one of the highest incidences of TB globally.^2^ An estimated 60% of new TB cases in SA are in persons with HIV (PWH).^3^ DILI is the most common severe adverse drug reaction (ADR) of anti-tuberculous therapy, occurring in 5% – 33% of adult patients.^4^ Anti-tuberculous drug-induced liver injury (AT-DILI) is a common reason for hospital admission and causes significant morbidity. A retrospective observational study of PWH and TB co-infection diagnosed with AT-DILI on admission to hospital in SA, showed an in-hospital and 3-month mortality of 27% and 35%, respectively.^5^

Risk factors for AT-DILI include co-infection with HIV, hepatitis B or C, pre-existing chronic liver disease, high alcohol intake, malnutrition, low body mass index (BMI), older age, female sex, slow N-acetyl transferase 2 (NAT-2) acetylator status,^4,6,7,8,9^ and co-administration of hepatotoxic medication. Most data on the risk of AT-DILI in PWH come from observational and retrospective cohort studies and estimates of incidence of hepatotoxicity range between 4% and 27%.^4^ These varied incidence rates can be partially explained by the variation in the definitions of AT-DILI. A specific risk factor for AT-DILI in children is low albumin.^10^

## Mechanisms and specific drugs

First-line anti-tuberculous drugs associated with hepatotoxicity include isoniazid (INH), RIF and pyrazinamide (PZA).^1^ These medicines can all cause hepatotoxicity individually or in combination, and there is overlap in the pattern of liver injury (Table 1). Anti-tuberculous DILI can be dose-related, idiosyncratic, or immune-mediated. All forms of AT-DILI can progress to fulminant liver failure and death, especially if the implicated drug is not stopped.

**TABLE 1:** The effect of anti-tuberculous drugs on the liver.

Drug	Pattern of injury	Mechanism of injury	Note
Pyrazinamide	Hepatocellular†	Drug extensively metabolised by liver. Dose-related injury suggesting direct toxic effect of drug or its metabolites.	May cause asymptomatic transient elevation in transaminases during hepatic adaptation.
Isoniazid	Hepatocellular† Onset varies from early (days to weeks) to late	Accumulation of toxic metabolites. Immune-mediated component.	May cause asymptomatic transient elevation in transaminases during hepatic adaptation. Risk increases with age. Rash, fever, and eosinophilia rarely.
Rifampicin	Hepatocellular†, cholestatic‡ or mixed Typically, within 1–6 weeks of initiation Associated jaundice	Drug extensively metabolised by liver. Direct toxic effect of RIF metabolites. Immune-mediated component	May cause asymptomatic transient elevation in transaminases during hepatic adaptation. Isolated increase in serum bilirubin can occur during first weeks of therapy. Fever, rash, arthralgias, and eosinophilia rarely.
Moxifloxacin, levofloxacin	Hepatocellular†, cholestatic‡ or mixed	Immune-mediated component	May cause asymptomatic transient elevation in transaminases during hepatic adaptation. Severe liver injury is rare. Fever, rash, and eosinophilia rarely.

RIF, rifampicin.

† , Hepatocellular picture includes raised hepatic transaminase enzymes with normal/minor abnormalities in alkaline phosphatase level.

‡ , Cholestatic picture includes raised bilirubin and alkaline phosphatase.

Anti-tuberculous drugs can also cause hepatic adaptation, which needs to be differentiated from AT-DILI. Hepatic adaptation can present as a transient, asymptomatic, low-grade elevation of transaminases, which settles on continuation of the drug and does not require intervention.^11^

Pyrazinamide can cause severe liver injury with a typically hepatocellular pattern of injury (an increase in transaminases with normal/minor abnormalities of alkaline phosphatase [ALP]). The mechanism of hepatotoxicity is not known. Liver injury is dose-related, suggesting a direct toxic effect of the drug and/or its metabolites.^12^

Isoniazid is also associated predominantly with transaminase elevation. The risk of hepatotoxicity from INH increases with age: it affects 0.5% of patients aged 20–35 years, 1.5% of those aged 35–50 years, and more than 3% of those over 50 years.^13,14^ Onset of liver injury can be early (within a few days to weeks) or late into therapy. Isoniazid toxicity is thought to be related to the accumulation of toxic metabolites. People with a slow NAT-2 acetylator status and cytochrome P450 2E1 (CYP2E1) polymorphisms are at increased risk for developing INH liver toxicity. Immune-mediated hypersensitivity reactions with rash, fever and eosinophilia can rarely occur, and rapid reoccurrence of injury on rechallenge suggests an immune-mediated component.^15^

RIF can cause mild, transient, asymptomatic elevations in transaminases in up to 20% of patients within the first 2 weeks of treatment initiation and represents hepatic adaptation.^16^ Isolated serum bilirubin increase without liver enzyme elevation has also been observed during the first weeks of therapy and does not represent liver injury but rather drug interference with bilirubin transport.^16^ RIF can also cause severe liver injury associated with jaundice, typically within 1 week to 6 weeks of initiation.^16^ The liver enzyme pattern can vary from the typical increase in transferase levels to a cholestatic picture (elevations of both bilirubin and ALP) to a mixed pattern. This is in contrast to INH and PZA, which are predominately hepatocellular.^16^ Extrahepatic manifestations such as fever, rash, arthralgias, and eosinophilia occur rarely. RIF is extensively metabolised by the liver and induces multiple hepatic enzymes. The mechanism of injury is thought to be due to direct toxic effects of RIF metabolites or to an immunologic reaction.^16^

Moxifloxacin and levofloxacin are used in the treatment of drug-resistant TB and in the background regimen for patients with AT-DILI on first-line TB treatment. A transient, mild, asymptomatic increase in hepatic transaminase enzymes, which resolves without discontinuation of therapy, occurs in 1% – 3% of patients receiving fluoroquinolones.^17^ This is more common with moxifloxacin than levofloxacin. Severe hepatocellular and cholestatic liver injuries have been rarely reported.^17^ The mechanism of action of the severe reactions is thought to be an immune-mediated hypersensitivity reaction and can present with fever, rash and eosinophilia.^17^

## Principles of managing adverse drug reactions

Managing ADRs involves several principles that aim to optimise patient safety and to mitigate harmful effects. These principles include risk assessment, patient education, prevention, close monitoring, early detection, reporting and documentation, and appropriate management.

To prevent ADRs, careful assessment of the patient should include a thorough medical history of allergies and past ADRs. Consideration should be given to potential drug interactions and contraindications. To enable early detection, close monitoring may be required. When an ADR occurs, the severity and potential risks associated with the reaction must be assessed. Management may involve discontinuation, dose adjustment, or switching to an alternative medication to ensure a complete regimen. The benefits and risks of continuing or discontinuing the suspected offending drug should be carefully evaluated and the duration of interruption minimised. Finally, it is essential that the patient be informed on how to take the medication and its side effects, and to be on the alert for any warning signs.

## Baseline and monitoring of liver function tests

Baseline LFTs are not recommended routinely for patients initiating anti-tuberculous therapy but are recommended if the patient has known risk factors associated with AT-DILI and may be available for some patients in whom LFTs were indicated for another reason. Abnormal LFTs are not a contraindication to start standard anti-tuberculous therapy; however, if a person has an abnormal alanine transaminase (ALT) or total bilirubin result at baseline, we recommend monitoring ALT and/or total bilirubin at least weekly until stable or improved. People with isolated elevations of aspartate transaminase (AST) or ALP/gamma-glutamyl transferase (GGT) do not require close laboratory monitoring. For patients with normal or unknown baseline LFTs, routine monitoring is not advised.

Liver function tests and international normalised ratio (INR) should be performed on any patient with visible jaundice or symptoms of hepatitis (e.g., nausea, vomiting, fatigue, right upper quadrant pain, anorexia, pale stools). LFTs and INR should also be requested on any patients presenting with a new rash while on anti-tuberculous treatment. Anti-tuberculous DILI can present as part of a hypersensitivity reaction with a rash suggestive of Stevens-Johnson Syndrome or Drug Reaction with Eosinophilia and Systemic Symptoms (DRESS).^18^ The management of a rash secondary to anti-tuberculous therapy is outside the scope of this guideline and specialist advice should be sought in these cases.

## Diagnosing anti-tuberculous drug-induced liver injury

A definitive diagnosis of AT-DILI requires the exclusion of alternative causes of raised liver enzymes and observation of the response to interrupting anti-tuberculous therapy. However, action needs to be taken before this process can be completed. Our suggested approach is to make a presumptive diagnosis of AT-DILI based on the pattern of abnormal liver enzymes with symptoms, then to confirm the AT-DILI diagnosis by exclusion of other competing diagnoses and further investigations, and based on the response to interrupting anti-tuberculous therapy.

### Presumptive diagnosis of anti-tuberculous drug-induced liver injury

A provisional diagnosis of AT-DILI should be made if a patient meets one of the following criteria while taking anti-tuberculous drugs:

- ALT > 120 IU/L ***or*** ALT > 3 × upper limit of normal (ULN) ***with***:
  ■ symptoms (vomiting, nausea, abdominal pain, encephalopathy, bleeding) ***or***
  ■ jaundice^4^ ***or***
  ■ total bilirubin > 40 µmol/L^19^
- ALT > 200 IU/L ***or*** ALT > 5 × ULN regardless of symptoms or bilirubin^4^
- ALT > 2 × baseline in patients with existing chronic liver disease or if the patient had a baseline ALT > 120 IU/L prior to anti-tuberculous therapy.

### Final diagnosis of anti-tuberculous drug-induced liver injury

A final diagnosis of AT-DILI should be made once other causes of liver dysfunction have been excluded. This should include a full history of all prescribed, over-the-counter, herbal or illicit drugs that have been consumed. LFT derangement usually improves on withdrawal of anti-tuberculous drugs; however, this may not be the case when the mechanism of injury is immune-allergic. In children, the most common cause of hepatitis is hepatitis A, and this should be excluded before proceeding to further investigations of alternative causes. In adults, it may be reasonable to test for hepatitis A, B and E concurrently. Testing for herpes simplex virus, cytomegalovirus and Epstein-Barr virus, either by serology or nucleic acid amplification testing, is not required in most cases.

### Abnormal liver function tests not fulfilling the definition of anti-tuberculous drug-induced liver injury

Abnormal LFTs that do not meet the criteria for AT-DILI as well as abnormal LFTs at baseline are not uncommon. Specific patterns of abnormalities that clinicians should be aware of that **do not** constitute AT-DILI, along with suggested strategies for investigation and management are listed below:

- *Isolated hyperbilirubinaemia* in a patient with no known liver disease is a total bilirubin > 40 µmol/L (> 2 × ULN) while other LFTs remain normal.^19,20,21,22^ Isolated hyperbilirubinaemia is usually benign and temporary; the only concern being cosmetic:
  ■ Continue anti-tuberculous therapy and repeat LFTs weekly. If total bilirubin remains elevated or increases to > 100 µmol/L (> 5 × ULN) discuss with an infectious diseases or hepatology specialist (Box 1). If hyperbilirubinaemia persists for more than a few weeks, a cholestatic hepatitis should be considered.
  ■ Once a downward trend has been established, the patient can be monitored monthly until resolution.
- *Cholestatic pattern* is characterised by a raised ALP and/or GGT, often with raised bilirubin in the absence of a significantly raised ALT (ALT < 120 IU/L [< 3 × ULN]).
  ■ The differential diagnosis for this pattern is broad. While TB drugs can cause a cholestatic pattern, other drugs like co-trimoxazole and fluconazole should be considered. Alternative causes such as granulomatous infiltration secondary to Immune Reconstitution Inflammatory Syndrome (IRIS) or biliary obstruction are more likely.
  ■ Continue anti-tuberculous therapy and investigate patients with abdominal ultrasound. If no cause is apparent or criteria for AT-DILI are met, discuss with an infectious diseases or hepatology specialist.^23,24^
- *ALT 120–200 IU/L (3–5 × ULN) with no symptoms*:
  ■ Continue anti-tuberculous therapy and check LFTs 1 week later: (1) If ALT is increasing, continue weekly monitoring until resolution or until AT-DILI criteria are met; (2) If ALT is static or decreasing, measure ALT after 1 week; if it continues to remain static or decrease, then measure monthly until resolution.
- *Baseline ALT > 120 IU/L (3 × ULN)*:
  ■ Start standard anti-tuberculous treatment and repeat LFTs after 2 weeks: (1) If ALT is increasing, continue to monitor LFTs weekly until either resolution or until AT-DILI criteria are fulfilled; (2) If ALT is static or decreasing, measure ALT after 1 week; if it continues to remain static or decrease, then measure monthly until resolution.

BOX 1 Contacts and resources for seeking reliable advice on anti-tuberculous DILI.

- University of Cape Town Medicines Information Centre (MIC) HIV & TB Hotline: 0800 212 506 or 021 406 6782. Alternatively send an SMS or ‘Please Call Me’ to 071 840 1572.
- Hepatology or infectious disease specialists can be contacted through the switchboard of any hospital with a hepatology or infectious disease service.
- Specialist adult infectious diseases advice is available by joining the Infectious Diseases Society of Southern Africa (IDSSA) WhatsApp group. Scan the QR code below using your phone.
- Specialist paediatric infectious diseases advice is available by joining the Southern African Society for Paediatric Infectious Diseases (SASPID) WhatsApp group. Scan the QR code below using your phone.

**Figure:**
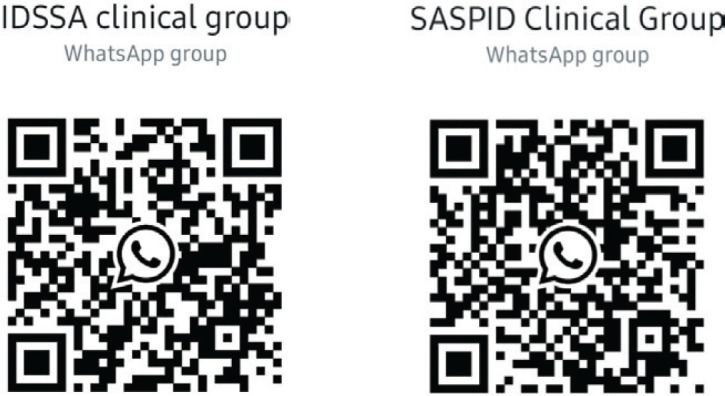

TB, tuberculosis.

As evidence is lacking, the guidance and frequency of monitoring of these patients is based on expert-opinion. Patient convenience is important to consider while keeping in mind that a prolonged follow-up interval may delay the diagnosis of liver dysfunction and therefore place the patient at risk for severe dysfunction and liver failure. Patients should be advised that if they experience new or worsening symptoms, they should return to the healthcare facility immediately.

## Guidance for patients meeting the definition of anti-tuberculous drug-induced liver injury

Once a presumptive diagnosis of AT-DILI has been made, the following steps are important:

- Review and confirm the evidence for the initial TB diagnosis: clinical, radiological, microbiological, and molecular investigations:
  ■ Note that a positive urine lipoarabinomannan (LAM) is suggestive of TB but can also be positive in alternative diagnoses such as non-tuberculous mycobacterial and nocardia infections.
- Perform additional investigations to verify the diagnosis of TB, if required. It is important to note that a TB culture result may have reverted to negative after a few weeks because of effective TB treatment.
- Review the likelihood of other diagnoses, such as community-acquired pneumonia.
- *If TB is microbiologically confirmed*:
  ■ Review the resistance profile.
  ■ Ensure the patient completes a full course of anti-tuberculous therapy.
- *If TB is not microbiologically confirmed*, consider the strength of the diagnosis on a case-by-case basis:
  ■ Assess the response to anti-tuberculous therapy such as weight gain, improvement in appetite, resolution of fever and night sweats, and improvement in respiratory symptoms.
  ■ This is especially important in children who are mostly clinically diagnosed.
  ■ If TB is possible or likely, ensure that the patient completes a full course of anti-tuberculous therapy.
  ■ If TB is unlikely, stop TB treatment and *do not* rechallenge.
  ■ If unsure, discuss with an infectious disease specialist.

### Severity assessment and criteria for admission

At the time of presentation with AT-DILI, all patients should have full LFTs, and an INR done. We propose the following simplified severity criteria for AT-DILI:

- *Mild*: no symptoms of hepatitis with INR < 1.5.
- *Moderate*: symptoms of hepatitis with INR < 1.5.
- *Severe*: INR > 1.5, regardless of symptoms.^19,20,25,26,27^

### Admission to hospital

In general, patients with AT-DILI should be admitted to hospital. One can consider monitoring mild disease as an outpatient provided the results are obtained within 24 h, and the patient can attend frequent and regular follow-ups for monitoring.

### Management of severe anti-tuberculous drug-induced liver injury

The management of patients with severe AT-DILI is complex and requires specialist input. In these cases, all drugs should be stopped, and clinicians should contact a specialist in either infectious diseases or hepatology for individualised advice on management, which is beyond the scope of this guideline.

### Management of mild or moderate anti-tuberculous drug-induced liver injury

The first step is to interrupt all TB medications as well as prophylactic co-trimoxazole, since co-trimoxazole can cause DILI with mostly a mixed picture. The decision to interrupt antiretroviral therapy (ART) should be individualised and we suggest the following approach:

- If on ART for < 6 months, consider interruption.
- If on an integrase inhibitor-based.
- ART regimen for > 6 months, consider continuing ART. Patients on protease-inhibitor-based ART should be switched to an integrase inhibitor-based regimen.
- If on efavirenz (EFV) for any duration, consider EFV as a cause of DILI and switch EFV to dolutegravir.

Fluconazole can also cause DILI; therefore, fluconazole used for secondary prophylaxis of cryptococcal meningitis can be stopped if the CD4 count is > 200 cells/mm^3^. If the patient is on fluconazole for the intensive or consolidation phase of treatment of cryptococcal meningitis *or* if the CD4 count < 200 cells/mm^3^, seek advice from an infectious disease specialist.

After eliciting a detailed medication history (including traditional medicines) consider the risks and benefits of withholding other hepatotoxic drugs on a case-by-case basis. If in doubt, seek specialist advice.

### Further investigations and supportive measures

In patients with mild or moderate AT-DILI, it is important to search for alternative causes and exacerbating factors. This includes viral hepatitis testing.

While multiple supportive measures are likely to be necessary in patients with severe AT-DILI, there is no indication for the routine use of vitamin K, lactulose, antibiotics, glucose monitoring or specific nutritional support for mild or moderate AT-DILI.

Routine testing for pregnancy in women of childbearing potential, with escalation to a higher level of care if positive, is strongly recommended due to the potential for more severe liver disease in pregnancy.

Abdominal ultrasound is usually unhelpful, except when the LFT abnormality is consistent with a cholestatic picture.

A recent study in SA^28^ randomised 102 patients with AT-DILI to receive either intravenous N-Acetylcysteine (NAC) or placebo. The primary outcome of time to ALT < 100 IU/L and the secondary outcome of mortality were similar between groups. However, those randomised to receive NAC had a significantly shorter median time to hospital discharge of 9 (6–15) days compared to 18 (10–25) days (hazard ratio [HR]: 1.73; 95% confidence interval [CI]: 1.13–2.65). This suggests that there may be some benefit to NAC in AT-DILI. We do not currently recommend the use of NAC in patients with mild or moderate DILI, although it may be prescribed on specialist advice to patients with severe AT-DILI.

### Use of anti-tuberculous therapy during recovery from anti-tuberculous drug-induced liver injury

There are limited data on the use of anti-tuberculous therapy during the recovery phase of AT-DILI. Decisions should be based on likely risk of disease progression in the absence of treatment. Factors to consider include the site of disease, burden of disease, clinical condition of the patient, and duration of TB treatment prior to discontinuation for AT-DILI.

As a general guide, we suggest that patients within the intensive phase of RIF-sensitive TB treatment receive anti-tuberculous therapy during the recovery from AT-DILI and that those within the continuation phase do not. Patients on continuation phase can receive anti-tuberculous therapy once the ALT is < 100 IU/L.

The suggested regimen during the recovery phase is standard doses of levofloxacin, ethambutol (EMB) and linezolid (LZD) (if the haemoglobin is < 8 g/dL use clofazimine or terizidone instead of linezolid).

### Monitoring patients during the recovery phase

In patients with an initial diagnosis of AT-DILI, ALT should be measured 2–3 times per week. A downward trend in ALT within a few days of stopping the drugs is in keeping with a presumed diagnosis of AT-DILI. In a study of patients hospitalised with AT-DILI, median time to ALT < 100 IU/L was 8 days (interquartile range [IQR]: 5–13).^28^ If ALT does not begin a downward trend in this time frame, alternative diagnoses should be considered, and specialist advice sought if necessary. Rechallenge of anti-tuberculous therapy is recommended when the ALT drops below 100 IU/L with total bilirubin on a downward trend (even if not normal), which often lags behind the ALT and is considered an unreliable marker of hepatocellular recovery.

### Rechallenging anti-tuberculous therapy

Studies suggest that between 20% and 90% of adult patients with AT-DILI can be rechallenged with anti-tuberculous therapy without reoccurrence.^29,30^ Reoccurrence of AT-DILI is strongly associated with the use of PZA as part of the rechallenge regimen.^29,31^

Specialist input is required to decide on drug rechallenge in patients with severe AT-DILI; however, patients with mild or moderate AT-DILI should typically be rechallenged with at least INH and RIF. Rechallenge of PZA is typically only recommended for TB meningitis (TBM) or if either INH or RIF is not tolerated. Box 2 details a suggested approach on how to rechallenge anti-tuberculous treatment and Table 2 indicates suggested regimens for patients who are not successfully rechallenged on all four anti-tuberculous drugs.

**TABLE 2:** Suggested regimens for patients who are not successfully rechallenged on all four anti-tuberculous drugs.

Drug not tolerated or not rechallenged	Suggested TB regimen/management
Pyrazinamide (PZA)	RIF + INH + EMB for 2 months then RIF + INH for 7 months.^32,33,34^
Isoniazid (INH)	RIF + EMB + PZA + LFX for 6 months. This can be extended for 9 months at clinician’s discretion.^35^
Rifampicin (RIF)	MDR-TB regimen according to latest South African guidelines.^35^
More than one core TB drug (RIF, INH, PZA)	Contact an ID specialist.

PZA, pyrazinamide; INH, isoniazid; RIF, rifampicin; EMB, ethambutol; LFX, levofloxacin; MDR-TB, multi-drug-resistant tuberculosis; TB, tuberculosis; ID, infectious disease.

BOX 2 Suggested approach to rechallenging anti-tuberculous treatment in adult PWH.

- **Day 1:**
  ■ Start INH 300mg daily.
  ■ Stop linezolid, clofazimine or terizidone (if prescribed) during the recovery phase.
- **Day 3:**
  ■ Check ALT: if increased, consider INH the cause of AT-DILI. If not, proceed.
- **Day 4:**
  ■ Add RIF 600 mg daily.
- **Day 7:**
  ■ Check ALT: if increased, consider RIF the most likely cause of AT-DILI.† If not, proceed.
- **Day 8:**
  ■ Add PZA if TBM or unsuccessful rechallenge of INH/RIF.
  ■ Stop levofloxacin if it is being used.
- **Day 10:**
  ■ Check ALT: if increased, consider PZA the most likely cause of AT-DILI.† If not, proceed.
- Check ALT weekly for 4 weeks after rechallenge.

INH, isoniazid; ALT, alanine transaminase; AT-DILI, anti-tuberculosis drug-induced liver injury; RIF, rifampicin; PZA, pyrazinamide; TBM, tuberculosis meningitis.

†, On rare occasions there may be a delayed reaction from a previously prescribed anti-tuberculous drug.

### Suggested regimens for patients who are not successfully rechallenged on all four anti-tuberculous drugs

Table 2 details the suggested regimens.

#### Special situations

**Tuberculosis meningitis:** Tuberculosis meningitis has the highest mortality of all forms of TB and can be more difficult to treat due to lower penetration of some drugs into the cerebrospinal fluid. Alternative regimens should be considered in patients with TBM who are intolerant of any first-line drugs (Table 3).

**TABLE 3:** Suggested regimens for patients with TBM who are intolerant of first-line anti-tuberculous drugs.

Drug not tolerated	Suggested TB regimen/management
Pyrazinamide (PZA)	RIF + INH + EMB + LFX for 12 months.^36,37^
Isoniazid (INH)	RIF + EMB + PZA + LFX for 6 months.^35^
Rifampicin (RIF)	MDR-TB central nervous system regimen according to latest South African guidelines.^35^
More than one core TB drug (RIF, INH, PZA)	Contact an ID specialist.

PZA, pyrazinamide; INH, isoniazid; RIF, rifampicin; EMB, ethambutol; LFX, levofloxacin; MDR-TB, multi-drug-resistant tuberculosis; TB, tuberculosis; ID, infectious disease; TBM, tuberculosis meningitis.

**Chronic liver disease:** An infectious disease or hepatology specialist should be contacted in cases of chronic liver disease.

**Pregnancy:** Pregnant patients can be treated in the same way as non-pregnant patients, but it is important to avoid pretomanid, ethionamide and aminoglycosides as these medications have not been shown to be safe in pregnancy.

### Rechallenging antiretroviral therapy and co-trimoxazole

If ART and co-trimoxazole was interrupted, they can sequentially be rechallenged at full dose around 2 weeks after the patient has become established on their new TB regimen.
